# Thickness-Dependent Light Transmittance and Temperature Rise in Dual-Cure Bioactive and Light-Cure Bulk-Fill Composite Resins

**DOI:** 10.3390/polym15132837

**Published:** 2023-06-27

**Authors:** Reema Alharbi, Eid Alharbi, Sanaa N. Al-Haj Ali, Ra’fat I. Farah

**Affiliations:** 1 Dental Intern, College of Dentistry, Qassim University, Al Mulayda 51452, Qassim, Saudi Arabia; 2 Department of Orthodontics and Pediatric Dentistry, College of Dentistry, Qassim University, Al Mulayda 51452, Qassim, Saudi Arabia; 3 Department of Prosthetic Dental Sciences, College of Dentistry, Qassim University, Al Mulayda 51452, Qassim, Saudi Arabia

**Keywords:** ACTIVA, bulk-fill, composite resin, dual-cure, light, light-cure, extended curing, temperature rise, thermocouples, transmittance

## Abstract

This study aimed to assess the light transmittance (T) and temperature increase through different increments of dual-cure bioactive bulk-fill restorative material (ACTIVA), light-cure bulk-fill, and conventional composite resin materials. Cylindrical specimens with a diameter of 8 mm and heights of 1, 2, 3, and 4 mm of ACTIVA, Tetric-N-Ceram bulk-fill (TBF), Filtek One bulk-fill (FBF), and Filtek Z250 (FZ) (n = 6 per group, 96 in total) were light-cured with a visible blue low-intensity light-emitting diode (LED) (650–800 mW/cm^2^ irradiance). T, and the temperature increase, were measured using an optical power meter and a digital thermometer during curing. The T mean values ranged between 0.012 and 0.239 (76.02 to 98.81% light attenuation), while the temperature rise mean values ranged between 9.02 and 20.80 °C. The parameters, including material type (partial eta squared (ηp^2^) = 0.284, *p* < 0.0001), thickness (ηp^2^ = 0.284, *p* < 0.0001), and their interaction (ηp^2^ = 0.185, *p* = 0.047), significantly affected the T values, whereas only the material type (ηp^2^ = 0.352, *p* = 0.047) affected the temperature rise values. The T and temperature rise mean values were highest in ACTIVA increments of 1-mm increments, in particular, showing the highest T mean values, followed by similar increments of TBF. A significantly higher T was found in 1-mm increments compared to thicker increments for all materials (*p* < 0.0001), and a significant positive correlation existed between T and temperature rise values (*r* = 0.348, *p* = 0.001). These findings show that the bioactive material ACTIVA and TBF allow for better T than the other materials, with ACTIVA recording a higher temperature rise. However, the large light attenuation observed for all materials, irrespective of thickness, suggests that curing in more than one location with a low-intensity LED is necessary to optimize the curing process. Furthermore, incremental filling of bulk-fill materials using a low intensity LED could be beneficial.

## 1. Introduction

Resin-based composite restorations were introduced in dentistry approximately 50 years ago as a method of restoring carious lesions in both primary and permanent teeth while providing an esthetically pleasing result [1,2]. However, to avoid problems, such as polymerization shrinkage, microleakage, and postoperative sensitivity, the material should be placed in increments of no more than 2 mm at a time [3]. To address this issue, DENTSPLY developed a new material called smart dentin replacement (SDR), which is a bulk-fill composite resin that can be applied in a single increment of up to 4 mm. SDR application could result in fewer overall layers required [4], which shortens chairside time and limits polymerization shrinkage [5]. 

Following the introduction of SDR, other flowable and high-viscosity bulk-fill composite materials have been developed. These materials are particularly useful for unco-operative children requiring procedures involving less chairside time [3,5]. However, the increased thickness of the bulk-fill composite resin materials can affect the passage of light through them during curing. During curing, the light that passes through the restoration is absorbed and scattered based on the size of the fillers and the refractive indices of the resin matrix and fillers. With increasing restoration depth, the light intensity is attenuated and its effectiveness is reduced, which reduces the light transmittance (T) [6]. T is an optical property defined as the ratio of transmitted irradiance to incident irradiance [7].

A relationship between the cavity depth, restoration thickness [8], type of light cure unit (LCU), light intensity generated, exposure time to the LCU, and heat generation [9] has been proposed. Heat generation occurs from the exothermic reaction during material setting as well as the heat generated by the LCU. The resultant temperature increase during polymerization can cause both reversible and irreversible pulpal inflammation or even necrosis [10]. Extensive research has been carried out on this subject, covering investigations on a diverse range of bulk-fill composite brands; however, varying and sometimes contradictory results exist. Studies have reported a temperature increase ranging from 0.8 to 13.3 °C [8,9,10,11,12,13,14,15,16]. Upon comparing bulk-fill materials with conventional materials, Kim et al. [13] found that higher temperatures are attained during curing, and the temperature rise tends to vary based on the region, with the maximum temperature occurring at the top center.

Recent developments have resulted in the creation of the first bioactive bulk-fill restorative material, ACTIVA BioACTIVE-RESTORATIVE, which mimics the physical and chemical properties of natural teeth and releases fluoride [17]. Bioactive materials have minimal polymerization shrinkage (<1.7%) and can stimulate remineralization when compared to conventional resin-based composite materials [18]. The manufacturers state that ACTIVA is moisture-friendly, making it particularly advantageous for dental treatment in children.

To date, no studies have investigated the T properties of ACTIVA or the degree of temperature increase within the material during curing, especially in comparison with other bulk-fill and conventional composite resin materials. Hence, addressing these points has potential implications for clinical practice, particularly in pediatric dentistry, as bioactive materials, such as ACTIVA, may offer advantages over conventional composite materials in terms of moisture tolerance and remineralization properties. Therefore, this study aimed to assess T and temperature rise through 1–4 mm increments of a dual-cure bioactive bulk-fill material (ACTIVA) with three other bulk-fill and conventional composite resin materials irradiated with a contemporary blue light-emitting diode (LED) LCU. The null hypothesis assumes similar T and temperature increase for all the materials and thicknesses. 

## 2. Materials and Methods

### 2.1. Specimen Preparation

In this in vitro study, four resin-based composites were analyzed, including three bulk-fill (two light-cured and one dual-cured) and one conventional composite resin. Table 1 provides information on the chemical composition, selected shade, and manufacturing companies of the materials assessed. Additionally, details regarding the chemical composition of ACTIVA are based on the findings of Francois et al. [19].

Cylindrical, hollow extra-firm silicone molds (Express TM VPS Bite Registration Material Putty, 3M ESPE, St. Paul, MN, USA) were fabricated to prepare the composite specimens. The molds were 1, 2, 3, and 4 mm thick and 25 mm in diameter. The central hollow region of the molds measured 8 mm in diameter. 

**Figure 1:**
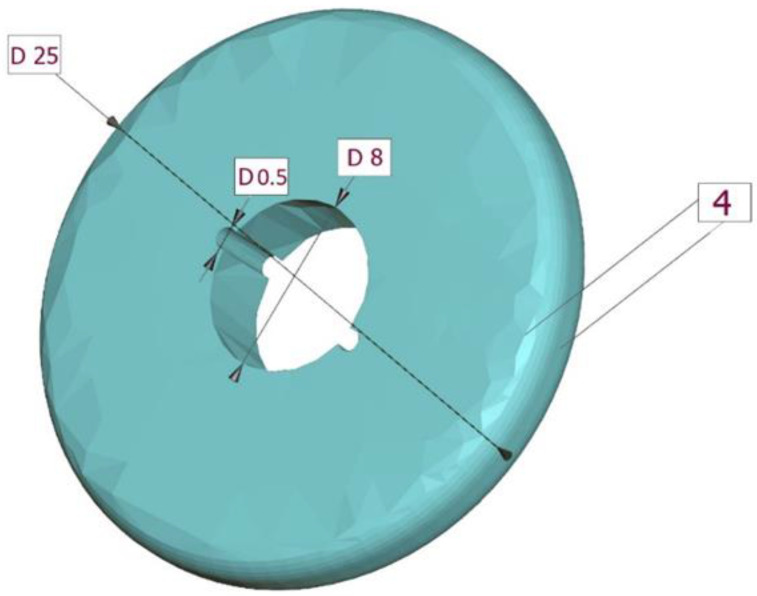
The 4-mm thick mold which was used in the study.

Two indentations 0.5 mm into the silicone material of the molds were prepared at two opposite directions of the wall of the hollow center to allow two k-type thermocouples to fit in those indentations (Figure 1), and connect to a digital thermometer (MS65514 digital thermometer, MASTECH^®^ group, Brea, CA, USA). 

A total of 96 specimens were fabricated using the silicone molds (24 from each material). All materials were filled in the molds as one increment using a plastic instrument over a transparent Mylar strip (Mylar Uni-strip, Dentsply Caulk, Milford, CT, USA). Likewise, the upper surface of the mold was covered with a Mylar strip during the curing process to prevent the formation of an oxygen-inhibited layer. 

For each material tested, six discs were fabricated in four different thicknesses (1–4 mm). Only with ACTIVA specimens, 20–30 s were allowed before curing the specimens according to the manufacturer’s recommendation. 

No power analysis was used to determine the sample size; however, we based the sample size on the work of Garoushi et al. [6], where five specimens for each combination of composite resin material and experimental condition were adequate. 

### 2.2. Light Transmittance Measurements

To determine the transmitted irradiance delivered to the bottom of each specimen at the assessed thickness, the light intensity (radiant power in mW) which passed through the mold during curing was measured using a digital optical power meter (Model 1830-C; Newport, CA, USA) connected to an optical sensor (818-SL/DB; Newport, CA, USA) with an OD3 attenuation filter according to the manufacturer’s instructions.

A fully charged visible blue LCU (LED-D, WOODPECKER, Guilin Woodpecker Medical Instrument Co., Guilin, China; 650–800 mW/cm^2^ radiant existence) was used to cure the specimens directly, and perpendicular to the Mylar strip which covered the top surface of the specimens. The curing duration was 40 s in full power mode and the average radiant power reading display on the optical power-meter screen was recorded. On the other hand, the reference LCU’s emitted radiant power was determined by placing the distal end of the 8 mm optical fiber of the LCU directly over the sensor at a distance of 0 mm. Three measurements were recorded and the average reading was obtained. 

The LCU’s emitted reference irradiance (incident irradiance) and transmitted irradiance (mW/cm²) through each specimen were calculated by dividing the radiant power measurements by 0.502 cm^2^, which corresponds to the circular surface area of the optical fiber for the LED device (cm^2^). Then, the ratio of the transmitted to the incident irradiance at each assessed thickness was calculated to allow for a direct comparison among materials and a quantification of T value. 

### 2.3. Temperature Rise

**Figure 2:**
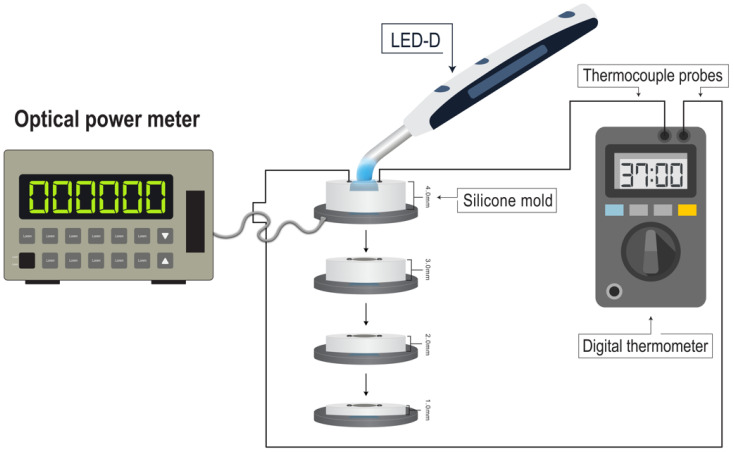
A schematic representation of the study apparatus.

While the specimens were being cured, the temperature rise in 40 s was measured by two K-type thermocouples fitted to the indentations of the silicone molds and connected to a digital thermometer. To facilitate heat conduction from all areas of the specimens and maximum temperature recording during curing, the thermocouples were lightly coated with a thermal compound (ARCTIC MX-4, ARCTIC Gmbh, Brunswick, Germany). Furthermore, to assess the heating effect of the LCU alone, the temperature rise was measured using the same thermocouples inside the molds in the same manner but without specimens. A schematic representation of the whole apparatus is given in Figure 2.

### 2.4. Statistical Analysis

Descriptive statistics for temperature rise and T values were generated. Mean values of temperature rise recorded by the thermocouples as well as T values were compared by a two-way ANOVA with factors of material and thickness. Bonferroni post hoc adjustment was used for multiple comparisons in all ANOVA models. Further, the relationship between the temperature rise and T values was determined using Pearson’s correlation coefficient. Statistical analysis was performed in SPSS version 20 (IBM, Armonk, NY, USA) with α = 0.05.

## 3. Results

### 3.1. Light Transmittance Values

The incident irradiance at the applied curing conditions was 770 mW/cm^2^ (reference radiant power 387 mW), while the transmitted irradiance values ranged between 9.19 (across 4-mm increments of FZ) and 184.61 mW/cm^2^ (across 1-mm increments of ACTIVA). This denotes a drop in irradiance reaching 76.02 to 98.81% when the light is passing across various thicknesses of the materials. 

A strong influence of both parameters—material type and thickness (*p* < 0.0001), as well as their interaction (*p* = 0.047)—was identified on the T values (Table 2). T values were highest in 1-mm ACTIVA increments followed by similar increments of TBF, being significantly different from 1-mm increments of FBF (*p* = 0.001) and FZ (*p* < 0.0001). With increasing thickness, T values only differed significantly among 2-mm increments of ACTIVA compared to similar increments of FZ (*p* = 0.009).

Among all materials, T values were significantly higher among 1-mm increments compared to thicker increments (*p* < 0.0001). Furthermore, 2-mm ACTIVA increments had significantly higher T than 3-mm (*p* = 0.048) and 4-mm (*p* = 0.013) increments. On the contrary, the T values among FZ composite increments differed significantly between 1-mm increments and 3-mm (*p* = 0.037) and 4-mm (*p* = 0.012) increments (Table 3). The independent effect of material type and thickness on mean T values is shown in Table 4.

### 3.2. Temperature Rise

Mean values of temperature rise as well as mean baseline and maximum temperature values are summarized in Table 5. Overall, the mean value for temperature rise of composite specimens was in the range between 9.02 and 20.80 °C. The empty molds measured temperature rise values between 4.7 °C (the 2-mm thick mold) and 8.4 °C (the 3-mm thick mold). The mean baseline temperature of the materials before curing ranged between 23.35 and 28.99 °C. The mean maximum temperatures recorded during curing ranged between 47.43 and 47.88 °C across ACTIVA 2–4-mm increments.

A strong influence of the parameter material was observed on temperature rise values, while neither the thickness of the specimens nor the interaction of material and thickness had a significant influence on temperature rise values (Table 2). The highest mean temperature rise values were among ACTIVA increments, irrespective of the thickness. Particularly among the 4-mm increments, the temperature rise values observed among ACTIVA increments were significantly higher than the rest of the increments (*p* < 0.05). Among the 2-mm increments, the values observed among ACTIVA increments were significantly higher than both FBF (*p* = 0.003) and FZ (*p* = 0.002) increments, and only FZ increments among the 3-mm increments (*p* = 0.022). The independent effect of composite material and thickness on mean temperature rise values is shown in Table 4.

**Figure 3:**
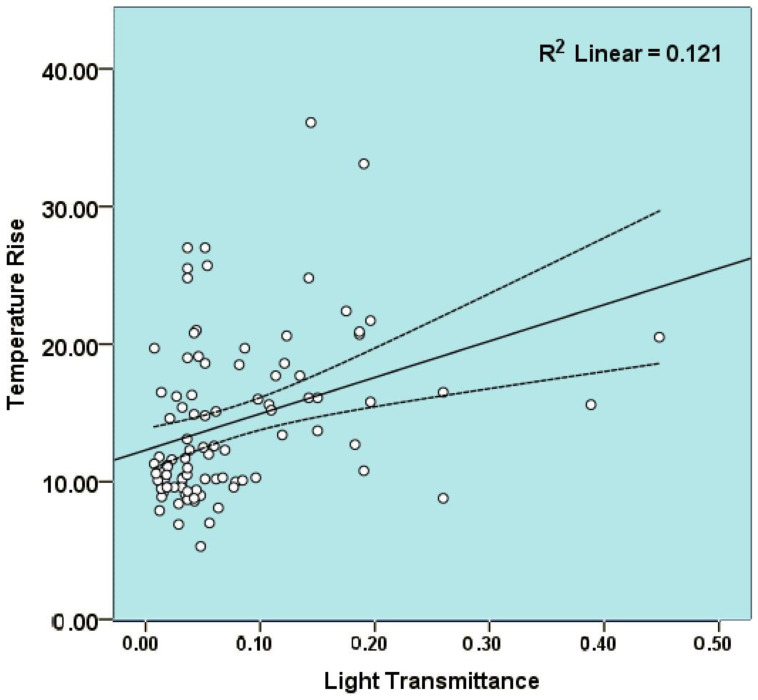
Relationship between light transmittance and temperature rise values. The middle lines show the line of best fit for the correlation and the two lateral lines show the 95% confidence intervals for the mean values.

When the relationship between temperature rise and T values was analyzed, there was a significant positive correlation (*r* = 0.348, *p* = 0.001) (Figure 3).

## 4. Discussion

This study compared the T and temperature rise of different materials in the most commonly used clinical shades for children. Light shades allow optimal light penetration compared with darker shades, which tend to absorb a greater amount of light owing to their opacity [3,20,21]. Consequently, the likelihood of the shade influencing the T results in the present study would be lower. A low-intensity LCU was used to cure all the assessed materials, as higher-intensity LCUs tend to generate more heat within bulk-fill composite resin materials, which would certainly influence the temperature rise measurements [11,14]. Furthermore, this was the recommended curing option as per the manufacturer instructions of ACTIVA.

Thermocouples were used to assess the temperature increase in this study, which is consistent with previous studies on bulk-fill composite resin materials. However, rather than measuring the temperature increase at the bottom of the specimens, we assessed the changes across the entire wall. Furthermore, we used a thermal compound to facilitate heat conduction [13].

Because T and the temperature rise during curing were significantly affected by the type of material, its thickness, and the interaction of the two factors, in the case of T in this study, the null hypothesis was rejected.

The temperature rise measurements ranged between 9.02 and 20.80 °C, and both the LCU and the polymerization exothermic reaction contributed to the temperature rise measurements since a rise of temperature reaching 8 °C was also noted in the empty molds.

Compared to the bulk-fill composite materials, the temperature rise in FZ was less, as previously noted [13]; however, our measurements were higher than those reported within bulk-fill composite materials (0.8 to 13.3 °C) [8,9,10,11,12,13,14,15,16]. Variations in the results of in vitro studies are expected owing to the differences in the materials investigated, characteristics of the LCU, and experimental set [22]. Pohto and Scheinin [23] previously stated that temperatures reaching 42–44 °C in rat teeth for a duration of 30 s were sufficient to stain the pulp, while arrest of the circulation occurred in the unexposed pulp at 46–60 °C when the exposure duration was two minutes. Temperatures exceeding 42 °C were detected among ACTIVA specimens in the present study as well as among TBF (1- and 4-mm increments). It is worth mentioning that values as high as 60.9 °C were also recorded among 2-mm increments of the hybrid composite material (Spectrum, Dentsply) using an infrared scanning system [22]. Similarly, Wang et al. [11] reported maximum temperatures reaching 55.9 °C using thermocouples on other bulk-fill composite materials. Consequently, in vitro studies are unable to determine the potential pulpal hazard [12], particularly in the absence of pulpal microcirculation, which can regulate temperature rise measurements [10,11], as well as the remaining dentin thickness, which can influence the amount of heat reaching the pulp owing to the low thermal conductivity of dentin [22] and periodontal tissues, which further contribute to the heat dissipation process.

In the present study, the temperature rise values were highest while curing ACTIVA (18–20 °C) with a statistically significant difference from the rest of the materials; however, increasing thickness did not show a clear pattern of either increase or decrease, unlike the rest of the materials, where the 1-mm increments showed a higher temperature rise than the thicker increments; however, the rise in temperature was insignificant. This was consistent with previous studies and was attributed to the insulating effect of the superficial composite layers, which caused a smaller temperature rise with increasing thickness [8,12,22,24].

ACTIVA is a dual-cure type of flowable bulk-fill composite material that is less viscous than other materials. It contains the least amount of filler (56%), which may be one reason for the temperature increase in this material. Previous studies have reported that the exothermic reaction during polymerization is proportional to the amount of filler; hence, flowable composite resins with low filler content show greater temperature increase than more viscous materials [9,10]. 

Other possible reasons are the curing duration (40 s) and location. All the materials in this study were cured for a longer duration than the manufacturers’ recommendations, except FBF, to increase the likelihood of optimal curing. Furthermore, curing was performed on the top surface, which is consistent with previous studies [5,7,20,24]. Jang et al. [25] noted that curing TBF for 20 s, according to the manufacturer’s recommendations, did not produce sufficient curing of the material with a low-intensity LED (700 mW/cm^2^). Some authors have recommended extending the manufacturer-recommended curing duration of the materials under investigation by as much as twice the value to compensate for light attenuation in the deep layers of the filling, as long as this does not generate excessive heat [20,26]. A curing duration of 30 s for FBF, TBF [12,27], and even FZ [24,27] was beneficial for increasing the depth of cure [27] and produced an acceptable temperature increase using a low-intensity LCU (800 mW/cm^2^ irradiance) [12]. Furthermore, T and surface roughness properties were previously assessed for these materials using a curing duration of 40 s with low-intensity (650 mW/cm^2^) and high-intensity LCU (≥1200 mW/cm^2^) [6,17]; moreover, the curing time did not affect the quality of the materials [17]. 

However, upon considering the T results in our study, significantly higher values were identified through ACTIVA followed by TBF specimens, while the least T was identified among FZ. Generally, bulk-fill composite resin materials are more translucent to blue light at all incremental thicknesses compared with conventional composites [28], and the lower filler content of ACTIVA is expected to increase its translucency. The T values in 1-mm increments were significantly higher than those in thicker increments for all the materials. This was expected after considering the findings from previous research [6,12]; however, light attenuation with increasing thickness was very large (76–98.8%).

Upon taking a closer look at the transmitted irradiance values in this study and converting them into radiant energies (irradiance × exposure time), the radiant energies of the various increments ranged from 0.37–7.38 J/cm^2^. Radiant energies required to adequately cure a composite layer are dependent on the material and reportedly vary between 6 and 36 J/cm^2^ [29]. While a minimum energy of ≥20 J/cm^2^ has been reported as the minimum dose required by Lima et al. [30] to polymerize 4-mm increments of bulk-fill resin-based composites, in this sense, extending the curing duration in three of the four materials using a low-intensity LED could not produce sufficient radiant energy, especially for 2–4 mm thick increments. According to the manufacturer’s recommendations, the FBF specimens did not produce the required radiant energy. Ilie and Furtos [7] previously stated that compensating the deficits in polymerization by additional light exposure could be challenging and may, in contrast, contribute to the temperature rise during curing. The positive, though weak, correlation between T and temperature rise supports this contribution. An R^2^ value of 0.121 implies that 12.1% of the variability in temperature rise was attributable to the changes in light transmittance and suggests that the LCU plays a role, as previously suggested [12]. The spatial heterogeneity of the light beam contributes to the discrepancy between the radiant energy received by the specimens and the radiant energy delivered through the curing unit tip [31]. Consequently, curing at more than one location whenever possible, particularly with a low-intensity LED, seems appropriate as some studies suggested [26,31], and perhaps splitting the curing duration into two cycles to generate less heat. Another option would be incremental filling bulk-fill composite materials.

It should be stressed that rest of the parameters, including angulation of the LED and distance from the specimens while curing, were controlled in this study despite not using a positioning jig to position the LED as employed by a few studies [6,7,11,24]. Moreover, many studies did not consider the positioning jig necessary as long as the light guide was in direct contact with, and perpendicular to, the top surface of the specimen [12,20]. Furthermore, in a clinical scenario, controlling all these factors is challenging, especially in children; consequently, polymerization in deep cavities could be concerning [11,26].

The present study had certain limitations that should be addressed. First, this study was an in vitro study conducted in a laboratory at room temperature, in contrast to clinical conditions where the baseline temperature of the oral cavity is 37 °C, and the pulp microcirculation, dental hard tissues, and periodontal tissues are prevalent, which could dissipate heat and limit the temperature rise. Second, we did not assess the degree of conversion, a parameter that predicts the depth of curing, in the cured specimens. Thus, further in vitro studies should be conducted on the T and temperature rise in bulk-fill composite materials with simulation of the pulp microcirculation and a baseline temperature of 37 °C, considering curing of more than one location as well as incremental filling.

## 5. Conclusions

Within the limitations of this study, some inferences can be drawn.

The type of bulk-fill composite material significantly affects both light transmittance and temperature rise during curing, whereas thickness only affects light transmittance, with the thinnest increment showing the best values. The ACTIVA and TBF materials exhibited better light transmittance than the other materials, with ACTIVA recording higher temperature increases. However, the large light attenuation observed for all materials, irrespective of thickness, suggests that curing in more than one location with a low-intensity LED is necessary to optimize the curing process and generate less heat.

## Figures and Tables

**Table 1:** The materials used in the study.

Composite Resin Trade Name and Shade	Classification	Composition	Recommended Curing Time and Intensity	Manufacturer
Tetric^®^ N-Ceram Bulk Fill (TBF)- IVA shade	Bulk Fill (Light-cure)	Resin: Dimethacrylates (19–20 weight %) Filler: Barium glass, ytterbium trifluoride (75–77% by weight)	20 s (≥500 mW/cm^2^)	Ivoclar Vivadent, Schaan, Liechtenstein Lot No.: Z02WLB
Filtek^™^ One Bulk Fill (FBF)- A2 shade	Bulk Fill (Light-cure)	Resin. *Bis-GMA*, *Bis-EMA*, *UDMA*, TEGDMA, *EDMAB* Filler: zirconia/silica, ytterbium trifluoride (65% by weight).	40 s 550–1000 mW/cm^2^	3 M ESPE, St. Paul, MN, USA Lot No.: 9177556
			20 s for > 1000 mW/cm^2^	
ACTIVA^™^ bioactive restorative- A1 shade	Bulk Fill (Dual-cure)	Resin: blend of diurethane and other methacrylates. Filler: Modified polyacrylic acid (44.6%), amorphous silica (6.7%), and sodium fluoride (0.75%) (56% by weight); reactive glass fillers (21.8% by weight).	20 s 550–1000 mW/cm^2^	Pulpdent, Watertown, MA, USA Lot No.: 211119
Filtek™ Z250 (FZ)- A1 shade	Conventional-micohybrid	Resin: *Bis-GMA*, *UDMA* and *Bis-EMA*. Filler: 77.5% by weight (0.01 μm to 3.5 μm particles).	20 s ≥400 mW/cm^2^	3 M ESPE, St. Paul, MN, USA Lot No.: NF27859

*Bis-GMA* Bisphenol A glycidyl dimethacrylate, *Bis-EMA* Ethoxylated bisphenol A dimetacrylate, *UDMA* Urethane dimethacrylate, TEGDMA: triethylene glycol dimethacrylate, *EDMAB*: ethyl 4-dimethyl aminobenzoate.

**Table 2:** Summary of results of two-way ANOVA (partial eta-squared values) as estimates of the effect size for the factors material type, thickness and their combinations on light transmittance and temperature rise values.

Factor	Dependent Variables			
	Temperature Rise		Light Transmittance	
	Partial Eta Squared	*p*-Value	Partial Eta Squared	*p*-Value
Material	0.352	<0.0001	0.284	<0.0001
Thickness	0.044	0.308	0.638	<0.0001
Material × Thickness	0.085	0.599	0.185	0.047

**Table 3:** Descriptive statistics of transmitted radiant power values, transmitted irradiance and light transmittance measurements.

Material	Thickness	Radiant Power (mW)	Transmitted Irradiance (mW/cm^2^)	Light Transmittance
TBF	1 mm	77.496 ± 19.190	154.252 ± 38.197	0.200 ± 0.049 ^A,a^
	2 mm	28.767 ± 20.771	57.258 ± 41.343	0.074 ± 0.054 ^b^
	3 mm	24.354 ± 5.697	48.474 ± 11.339	0.063 ± 0.015 ^c^
	4 mm	13.618 ± 1.675	27.107 ± 3.334	0.035 ± 0.004 ^d^
FBF	1 mm	52.325 ± 17.093	104.151 ± 34.022	0.135 ± 0.044 ^B,a^
	2 mm	22.443 ± 4.738	44.671 ± 9.432	0.058 ± 0.012 ^b^
	3 mm	15.725 ± 3.721	31.299 ± 7.406	0.041 ± 0.009 ^c^
	4 mm	6.385 ± 1.464	12.709 ± 2.913	0.017 ± 0.003 ^d^
ACTIVA	1 mm	92.747 ± 55.433	184.608 ± 110.337	0.239 ± 0.143 ^A,a^
	2 mm	44.638 ± 17.403	88.849 ± 34.639	0.115 ± 0.044 ^A,b^
	3 mm	16.739 ± 4.793	33.318 ± 9.539	0.043 ± 0.012 ^c^
	4 mm	12.275 ± 4.996	24.433 ± 9.944	0.0317 ± 0.012 ^d^
FZ	1 mm	37.446 ± 20.685	74.534 ± 41.172	0.097 ± 0.053 ^C,a^
	2 mm	10.951 ± 3.234	21.798 ± 6.437	0.028 ± 0.008 ^B^
	3 mm	8.556 ± 3.866	17.029 ± 7.695	0.022 ± 0.009 ^b^
	4 mm	4.617 ± 1.509	9.191 ± 3.005	0.012 ± 0.003 ^c^

Different letters indicate significant differences in light transmittance values within the column, small letters indicate significant differences across thicknesses of the same material while capital letters indicate significant differences between materials; TBF: Tetric-N-Ceram bulk-fill, FBF: Filtek one bulk-fill, FZ: Filtek Z250.

**Table 4:** Independent effect of composite material and thickness on mean temperature rise and light transmittance values.

Light transmittance	Amongst resin composites	ACTIVA ^a^ > FBF ^c^, FZ ^d^ TBF ^b^ > FZ ^d^
	Amongst various thicknesses	1 mm ^a^ > 2 ^b^, 3 ^c^, and 4 ^d^ mm 2 mm ^b^ > 4 mm ^d^
Temperature rise	Amongst resin composites	ACTIVA ^a^ > TBF ^b^, FBF ^c^, FZ ^d^
	Amongst various thicknesses	The differences were insignificant

> indicates statistical significance in mean light transmittance or temperature rise values; Lowercase letters represent mean values in descending order (e.g “a”: maximum mean value); TBF: Tetric-N-Ceram bulk-fill, FBF: Filtek one bulk-fill, FZ: Filtek Z250.

**Table 5:** Descriptive statistics of temperature rise values, and baseline to maximum mean temperature values.

Thickness		Material			
		TBF	FBF	ACTIVA	FZ
1mm	Baseline to maximum (mean)	24.65–40.90	23.35–37.25	24.58–43.00	26.00–41.28
	Temperature rise (mean ± SD)	16.25 ± 4.72	13.90 ± 3.13	18.42 ± 3.09	15.28 ± 8.81
2 mm	Baseline to maximum (mean)	27.15–43.60	26.83–36.02	27.80–47.43	28.77–37.78
	Temperature rise (mean ± SD)	16.45 ± 11.42	9.18b ± 3.31 ^a^	19.63 ± 3.55 ^b^	9.02 ± 1.36 ^c^
3 mm	Baseline to maximum (mean)	27.35–41.92	24.98–36.55	28.78–47.63	26.18–36.50
	Temperature rise (mean ± SD)	14.57 ± 4.49	11.57 ± 1.30	18.85 ± 4.50 ^a^	10.32 ± 0.94 ^b^
4 mm	Baseline to maximum (mean)	28.99–42.02	25.22–36.30	27.08–47.88	23.70–34.18
	Temperature rise (mean ± SD)	13.03 ± 6.60 ^a^	11.08 ± 2.97 ^b^	20.80 ± 4.41 ^c^	10.48 ± 0.93 ^d^

Different letters indicate the significant differences in temperature rise values within rows; TBF: Tetric-N-Ceram bulk-fill, FBF: Filtek one bulk-fill, FZ: Filtek Z250.

## Data Availability

The datasets used and/or analyzed during the current study are available from the corresponding author upon reasonable request.

## References

[1] American Academy of Pediatric Dentistry (2018). Pediatric Restorative Dentistry. Pediatr. Dent..

[2] Al-Haj Ali S.N., Alsulaim H.N., Albarrak M.I., Farah R.I. (2021). Spectrophotometric comparison of color stability of microhybrid and nanocomposites following exposure to common soft drinks among adolescents: An in vitro study. Eur. Arch. Paediatr. Dent..

[3] Bayrak G.D., Yaman-Dosdogru E., Selvi-Kuvvetli S. (2022). The Effect of Two Different Light-Curing Units and Curing Times on Bulk-Fill Restorative Materials. Polymers.

[4] (2014). The first bulk-fill composite. Br. Dent. J..

[5] Oter B., Deniz K., Cehreli S.B. (2018). Preliminary data on clinical performance of bulk-fill restorations in primary molars. Niger. J. Clin. Pract..

[6] Garoushi S., Vallittu P., Shinya A., Lassila L. (2016). Influence of increment thickness on light transmission, degree of conversion and micro hardness of bulk fill composites. Odontology.

[7] Ilie N., Furtos G. (2020). A Comparative Study of Light Transmission by Various Dental Restorative Materials and the Tooth Structure. Oper. Dent..

[8] Mousavinasab S.M., Taromi Z., Zajkani E. (2020). Thermal rise during photopolymerization and degree of conversion of bulk fill and conventional resin composites. Dent. Res. J..

[9] Altan H., Göztas Z., Arslanoglu Z. (2018). Bulk-Fill Restorative Materials in Primary Tooth: An Intrapulpal Temperature Changes Study. Contemp. Clin. Dent..

[10] Yasa E., Atalayin C., Karacolak G., Sari T., Turkun L.S. (2017). Intrapulpal temperature changes during curing of different bulk-fill restorative materials. Dent. Mater. J..

[11] Wang W.J., Grymak A., Waddell J.N., Choi J.J.E. (2021). The effect of light curing intensity on bulk-fill composite resins: Heat generation and chemomechanical properties. Biomater. Investig. Dent..

[12] Par M., Repusic I., Skenderovic H., Milat O., Spajic J., Tarle Z. (2019). The effects of extended curing time and radiant energy on microhardness and temperature rise of conventional and bulk-fill resin composites. Clin. Oral Investig..

[13] Kim R.J., Son S.A., Hwang J.Y., Lee I.B., Seo D.G. (2015). Comparison of photopolymerization temperature increases in internal and external positions of composite and tooth cavities in real time: Incremental fillings of microhybrid composite vs. bulk filling of bulk fill composite. J. Dent..

[14] Odum N.C., Ross J.T., Citrin N.S., Tantbirojn D., Versluis A. (2023). Fast Curing with High-power Curing Lights Affects Depth of Cure and Post-gel Shrinkage and Increases Temperature in Bulk-fill Composites. Oper. Dent..

[15] Santis R., Lodato V., Gallicchio V., Prisco D., Riccitiello F., Rengo S., Rengo C. (2020). Cuspal Deflection and Temperature Rise of MOD Cavities Restored through the Bulk-Fill and Incremental Layering Techniques Using Flowable and Packable Bulk-Fill Composites. Materials.

[16] Nilsen B.W., Mouhat M., Haukland T., Örtengren U.T., Mercer J.B. (2020). Heat Development in the Pulp Chamber During Curing Process of Resin-Based Composite Using Multi-Wave LED Light Curing Unit. Clin. Cosmet. Investig. Dent..

[17] Alkhudhairy F., Vohra F., Naseem M., Ahmad Z.H. (2019). Adhesive bond integrity of dentin conditioned by photobiomodulation and bonded to bioactive restorative material. Photodiagn. Photodyn. Ther..

[18] Alrahlah A. (2018). Diametral Tensile Strength, Flexural Strength, and Surface Microhardness of Bioactive Bulk Fill Restorative. J. Contemp. Dent. Pract..

[19] Francois P., Fouquet V., Attal J.P., Dursun E. (2020). Commercially Available Fluoride-Releasing Restorative Materials: A Review and a Proposal for Classification. Materials.

[20] De Mendonça B.C., Soto-Montero J.R., de Castro E.F., Kury M., Cavalli V., Rueggeberg F.A., Giannini M. (2022). Effect of extended light ACTIVAtion and increment thickness on physical properties of conventional and bulk-filled resin-based composites. Clin. Oral Investig..

[21] Guiraldo R.D., Consani S., Consani R.L., Berger S.B., Mendes W.B., Sinhoreti M.A. (2009). Light energy transmission through composite influenced by material shades. Bull. Tokyo Dent. Coll..

[22] Al-Qudah A.A., Mitchell C.A., Biagioni P.A., Hussey D.L. (2007). Effect of composite shade, increment thickness and curing light on temperature rise during photocuring. J. Dent..

[23] Pohto M., Scheinin A. (1958). Microscopic Observations on Living Dental Pulp II. The Effect of Thermal Irritants on the Circulation of the Pulp in the Lower Rat Incisor. Acta Odontol. Scand..

[24] Ilie N. (2017). Impact of light transmittance mode on polymerisation kinetics in bulk-fill resin-based composites. J. Dent..

[25] Jang J.H., Park S.H., Hwang I.N. (2015). Polymerization shrinkage and depth of cure of bulk-fill resin composites and highly filled flowable resin. Oper. Dent..

[26] De Cássia Romano B., Soto-Montero J., Rueggeberg F.A., Giannini M. (2020). Effects of extending duration of exposure to curing light and different measurement methods on depth-of-cure analyses of conventional and bulk-fill composites. Eur. J. Oral Sci..

[27] Zorzin J., Maier E., Harre S., Fey T., Belli R., Lohbauer U., Petschelt A., Taschner M. (2015). Bulk-fill resin composites: Polymerization properties and extended light curing. Dent. Mater..

[28] Bucuta S., Ilie N. (2014). Light transmittance and micro-mechanical properties of bulk fill vs. conventional resin based composites. Clin. Oral Investig..

[29] Santini A., Miletic V., Swift M.D., Bradley M. (2012). Degree of conversion and microhardness of TPO-containing resin-based composites cured by polywave and monowave LED units. J. Dent..

[30] Lima R.B.W., Troconis C.C.M., Moreno M.B.P., Murillo-Gómez F., De Goes M.F. (2018). Depth of cure of bulk fill resin composites: A systematic review. J. Esthet. Restor. Dent..

[31] Shimokawa C.A.K., Turbino M.L., Giannini M., Braga R.R., Price R.B. (2018). Effect of light curing units on the polymerization of bulk fill resin-based composites. Dent. Mater..

